# Determination of Active Ingredients, Mineral Composition and Antioxidant Properties of Hydroalcoholic Macerates of *Vinca minor* L. Plant from the Dobrogea Area

**DOI:** 10.3390/molecules28155667

**Published:** 2023-07-26

**Authors:** Ana-Maria Neculai, Gabriela Stanciu, Magdalena Mititelu

**Affiliations:** 1 Department of Biochemistry, Faculty of Medicine, Ovidius University of Constanta, Street Universitatii, No. 1, 900470 Constanta, Romania; anamneculai89@gmail.com; 2 Department of Chemistry and Chemical Engineering, Ovidius University of Constanta, 124 Mamaia Blvd., 900527 Constanta, Romania; 3 Clinical Laboratory and Food Hygiene Department, Faculty of Pharmacy, “Carol Davila” University of Medicine and Pharmacy, 3-6 Traian Vuia Street, 020956 Bucharest, Romania

**Keywords:** indole alkaloids, *Vinca minor* macerates, DPPH Radical Scavenging test, HPLC, metal concentration, photo-chemiluminescence, phytocompounds

## Abstract

In recent decades, new alternative therapies using drugs containing active ingredients of natural origin have been a hot topic for medical research. Based on the confirmed therapeutic potential of the *Vinca minor* plant, considered in the specialized literature to be of pharmaceutical interest, the purpose of this study is to determine the chemical and mineral composition of the *Vinca minor* plant grown in the Dobrogea area, with a view to its use in the formulation of dermal preparations. For this purpose, plant materials were collected from the mentioned area and hydroalcoholic macerates of different concentrations were obtained: 40%, 70% and 96% from leaves (F40, F70, F96) and stems (T40, T70, T96) of *Vinca minor* plant to determine the optimal extraction solvent. The hydroalcoholic macerates were analyzed via the HPLC method for the identification and quantification of the main bioactive compounds, and two methods were used to evaluate their antioxidant properties: the DPPH radical scavenging test and the photochemiluminescence method. HPLC analysis showed the presence of four indole alkaloids: vincamine, 1,2-dehydroaspidospermidine, vincaminoreine and eburnamonine. Vincamine was the alkaloid found in the highest concentration in Vinca leaves (2.459 ± 0.035 mg/100 g d.w.). The antioxidant activity of *Vinca minor* hydroalcoholic macerates showed values between 737.626–1123.500 mg GAE/100 g d.w (DPPH test) and 77.439–187.817 mg TE/100 g d.w (photochemiluminescence method). The concentrations of toxic metals Cd, Cu, Ni, Pb in dried leaves and stems of *Vinca minor*, determined by AAS, were below detection limits.

## 1. Introduction

Alkaloids are an important class of natural products with wide distribution as well as chemical, biological and medical significance. *Apocynaceae* is one of the most important families containing a large number of alkaloids, of which 2664 have been isolated and characterized [1]. Due to their cytotoxic and hypoglycemic effect they were previously used in the treatment of diabetes, hypertension or as disinfectants. Today, they are mainly used in the treatment of cancer. In metaphase, due to their binding to tubulin, they affect microtubule function, stopping cell division and ultimately cell apoptosis [2,3].

There are about seven species of *Vinca* (*Apocynaceae*) in the world. The most representative medical plant in the *Apocynaceae* family is *Vinca minor* [4,5]. *Vinca minor* is a perennial plant native to northern Spain and western France, which has naturalized in several areas, including the Dobrogea area of Romania. In folk medicine, *Vinca minor* is known for its sedative, hypotensive, antidiabetic effects and also for treating circulatory disorders or promoting cerebral metabolism [5]. 

The specialized literature has identified approximately 50 alkaloids with indolic structures, and vincamine is the alkaloid found in the highest concentrations in *Vinca minor* plants [6,7]. In addition to alkaloids, other important natural compounds found in *Vinca minor* include flavonoids, phenolic acids, carotenoids, and amino acids [8,9,10,11,12]. Vincamine has modulatory effects on brain circulation and neuronal homeostasis, as well as anti-hypoxic and neuroprotective properties. Vincamine is used for the prevention and treatment of cerebrovascular insufficiencies and disorders by increasing cerebral blood flow, oxygen consumption, and glucose utilization [13,14]. Externally, it exhibits wound-healing effects by accelerating the healing process of injuries [15]. Despite the beneficial effects of this plant, drug resistance is a major clinical problem [14]. Therefore, the search for new compounds with therapeutic effects has proven to be very important. 

Considering the data in the literature, this study aims to promote these natural resources for therapeutic purposes. In order to determine the optimal formula for extracting the active principles from *Vinca minor*, the aerial parts of the plant (leaves and stems) were analyzed separately using ethyl alcohol of different concentrations: 40%, 70%, and 96% as solvent. For the identification and quantification of the principal bioactive compounds of the hydroalcoholic macerates obtained from *Vinca minor*, the HPLC method was used and the antioxidant capacity was evaluated via two methods: a DPPH radical scavenging test and the photochemiluminescence method. The mineral composition of dried leaves and stems of *Vinca minor* was also analyzed to determine the possible degree of toxicity.

The aim of these investigations was to evaluate the possibility of using the plant in different pharmaceutical formulations and especially in dermal preparations with pharmaceutical potential.

## 2. Results

### 2.1. Separation and Determination of Indole Ring Alkaloids in Vinca minor via HPLC

Table 1 shows the results of HPLC analysis for leaf samples as well as *Vinca minor* stems. Capacity factors and retention times of the studied alkaloids are presented.

**Figure 1:**
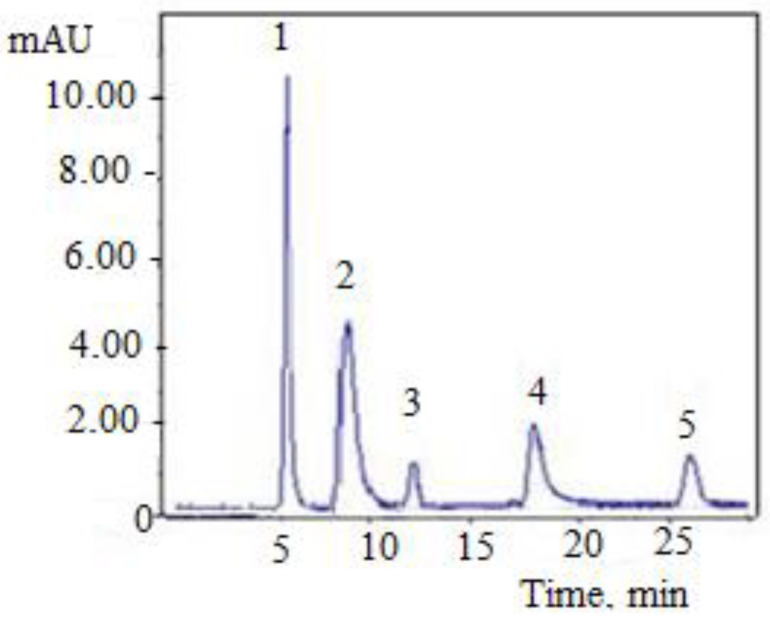
Chromatogram for the leaf of *Vinca minor* plant: 1—paclitaxel; 2—vincamine; 3—eburnamonin; 4—1,2-dehydroaspidospermidine; 5—vincaminorein.

**Figure 2:**
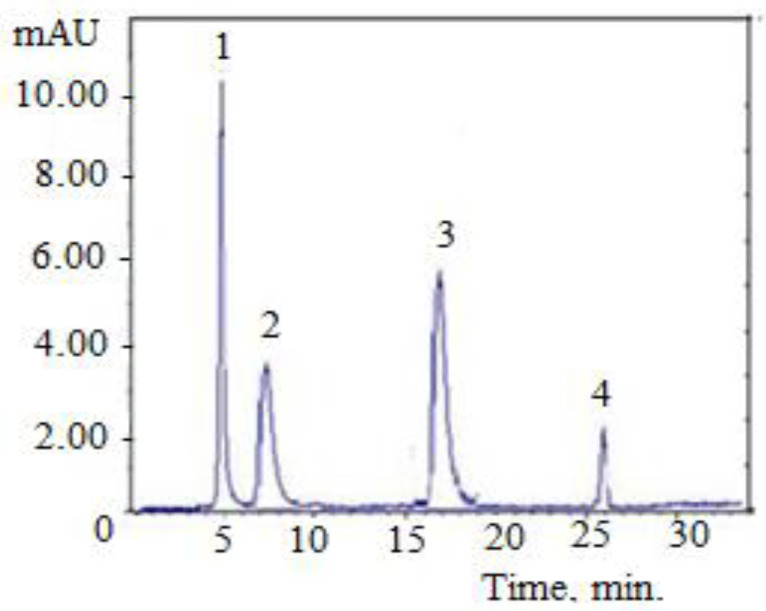
Chromatogram for the stem of *Vinca minor* plant: 1—paclitaxel; 2—vincamine; 3—1,2-dehydroaspidospermidine; 4—vincaminorein.

Figure 1 and Figure 2 show the chromatograms of extracts obtained from the leaf and stem of the *Vinca minor* plant.

### 2.2. Mineral Composition of Vinca minor Plant

The results obtained from the analysis of the mineral composition of the *Vinca minor* plant are presented in Table 2.

### 2.3. Antioxidant Activity of Vinca minor Hydroalcoholic Macerates

#### 2.3.1. DPPH Radical Scavenging Test

The antioxidant activity of *Vinca minor* hydroalcoholic macerates, evaluated via the DPPH radical scavenging test, is shown in Table 3.

#### 2.3.2. Photochemiluminescence Method

Total antioxidant capacity of *Vinca minor* hydroalcoholic macerates in 40:60 (*v*:*v*), 70:30 (*v*:*v*), and 96:4 (*v*:*v*) ethanol, compared to Trolox^®^ standard, was quantified according to the ACL procedure (Analytik Jena AG) for stock solution and for different dilutions of stock solution 1:10, 1:100, 1:200. The quantitative results, expressed in mg TE/100 g d.w., are presented in Table 4.

## 3. Discussion

The HPLC results indicate that the most predominant alkaloid in *Vinca minor* is vincamine (Figure 1 and Figure 2). Following HPLC determinations (Table 1), vincamine shows the highest concentration (2.459 mg/100 g d.w.) in leaves. These results are in agreement with the specialized literature [16]. The alkaloid eburnamonine was detected only in the leaves of the *Vinca minor* plant in a 3-fold smaller amount (0.803 mg/100 g d.w.). The alkaloid 1,2-dehydroaspidospermidine was found in both leaves and stems, with a greater amount in stems, 1.635 mg/100 g d.w. compared to 0.898 mg/100 g d.w. in the leaves. The alkaloid vincaminoreine was detected in the leaves and stems of the plant, *Vinca minor*, with a higher content in the leaves (1.064 mg/100 g d.w.) than in stems (0.285 mg/100 g d.w.).

Vincamine has vasodilatory and hypotensive effects, with a predominant action on cerebral circulation. Externally, vincamine has a healing effect [15]. Eburnane-type alkaloids (eburnamonin), aspidospermine-type alkaloids (1,2-Dehydroaspidospermidine) and quebracharnine-type alkaloids (vincaminorein) were also identified in the studied plant and known for their antitumor properties, being used in the treatment of cancer [12,13]. To ensure the quality and safety of herbal pharmaceutical preparations, it is necessary to evaluate the mineral composition of the studied plants. Table 2 shows the mean values of three determinations for the metals analyzed in the leaves and stems of the *Vinca minor* plant. The main (Ca, Mg and Na) and secondary (Fe and Mn) elements showed significant concentration values, while the concentrations of toxic elements (Cd, Cu, Ni, Pb) were below the detection limits. Calcium is an essential element for all plants, but plants differ widely in the amounts of calcium they require [17]. Of all the metals analyzed, calcium had the highest concentrations (4896 ± 0.52 and 25,640 ± 3.14), both in leaves and stems, of all the trace elements analyzed. Magnesium is a component of chlorophyll molecules and is therefore essential for photosynthesis [18]. In this study (Table 2), magnesium levels ranged from 1436.2 mg/kg for leaf to 871.4 mg/kg for stem. Although sodium is not essential for plants, it is useful in many conditions, especially potassium deficiency. Therefore, it can be considered a functional nutrient [19]. Sodium concentration in dry plant material varied from 874.20 mg/kg in leaves to 1293.2 mg/kg in stems. Iron and manganese play important roles in plant growth and development, but often compete for uptake, as an excess amount of one of these micronutrients makes the other less available to plant roots [20]. The iron concentration varied from 192.34 mg/kg to 620.80 mg/kg, with the maximum in the leaves of the studied plant. Manganese varied from 92.34 mg/kg to 296 mg/kg, with the maximum in the leaves. Zinc is essential for plants and plays an important role in cell division, protein synthesis and auxin production [19]. Zinc concentration was higher in leaves: 64.85 mg/kg.

The antioxidant activity of hydroalcoholic macerates from Vinca leaves, evaluated via the DPPH radical scavenging test, showed values between 768 mg GAE/100 g d.w. and 1186 mg GAE/100 g d.w. (Table 3). Regarding hydroalcoholic macerates from Vinca stems, the DPPH test recorded values between 737.626 mg GAE/100 g d.w. and 994.875 mg GAE/100 g d.w. The hydroalcoholic macerations obtained with 70% ethyl alcohol presented an antioxidant capacity close to that of those with 40% ethyl alcohol and superior to those with 96% ethyl alcohol. These results indicate 70% ethyl alcohol as the optimal solvent for the extraction of bioactive compounds with antioxidant activity. The results obtained are consistent with our previous research on the determination of the total content of polyphenolic compounds in *Vinca minor* hydroalcoholic macerates, known as compounds with strong antioxidant activity [21].

Analyzing the results obtained with the photochemiluminescence method, it was found that the stock solution generated good antioxidant activity in the case of the hydroalcoholic macerates obtained from the leaves and stems of the *Vinca minor* plant with 40% and 70% ethanol. In the case of T96 and F96 hydroalcoholic macerates, the stock solution shows inhibition, but the TEAC result does not fit the standard curve, but the 100-fold diluted samples have good antioxidant capacity. The hydroalcoholic macerates of strains T40 and T70 show a very close antioxidant activity of 187.367 mg TE/100 g d.w. and 187.267 mg TE/100 g d.w., respectively. The same was found in the case of hydroalcoholic macerates from F40 and F70 leaves, for which 187.717 mg TE/100 g d.w., respectively 187.817 mg TE/100 g d.w. were determined. Dilution of the sample at molar ratios of 1:10, 1:100, and 1:200, respectively, and the minimum working volume of 5 μL were observed to decrease the maximum inhibition of free radicals due to the increase in the amount of TEAC (mg TE/100 g d.w.).

The results are in agreement with those determined via the DPPH test. The analysis of the antioxidant activity highlights the possibility of optimal exploitation of the antioxidant potential in the form of hydroalcoholic macerates from different parts of the plant for further processing in different effective pharmaceutical formulations.

## 4. Materials and Methods

### 4.1. Plant Material and Extract Preparation

**Figure 3:**
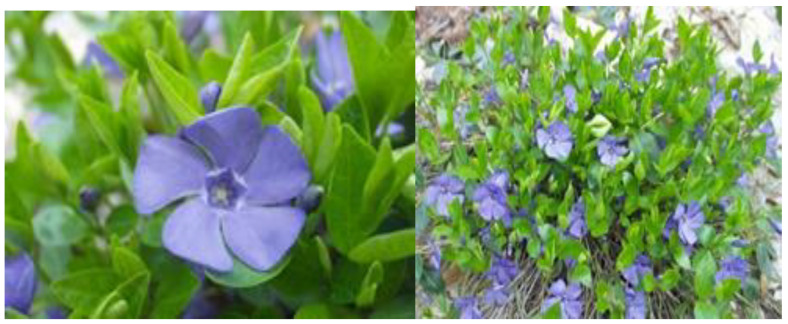
*Vinca minor* L. collected from Siutghiol Lake in the Dobrogea area (44°16′12″ N; 28°33′36″ E), Romania.

The aerial parts (leaves and stems) of *Vinca minor* L. plant were collected during the flowering period in May 2022. *Vinca minor* L. is a perennial plant that grows in the spontaneous flora of Romania and was harvested, from the edge of the Siutghiol Lake in the Ovidiu area, located in the Dobrogea region, (44°16′12″ N; 28°33′36″ E), Romania (Figure 3).

The plant products were washed thoroughly with distilled water and dried naturally on nets located in a well-ventilated room. Following this, dried plant material was ground into a fine powder using a laboratory mill. The preparation process of the hydroalcoholic macerates involved the chopping of 50 g of plant product obtained from leaves and stems of *Vinca minor* plant, over which ethyl alcohol was added in concentration of 40%, 70%, 96%, up to 500 mL, respectively (ratio 1:10). The macerates were left for 10 days under optimal conditions, away from light and humidity, in a cool place. During the 10-day period, the extracts were carefully observed and shaken 2–3 times daily. At the end, they were filtered through cotton filters to separate them from the plant material. The liquid collected during filtration was stored in sterile, dark containers.

### 4.2. Phytochemical Analyses of the Vinca minor Extracts

#### 4.2.1. HPLC Method

All reagents used were of high analytical purity (intended for HPLC method): methanol (Sigma-Aldrich, Germany), chloroform, hydrochloric acid, ammonia, and triethylamine (Merck, Darmstadt, Germany). Deionised water was used. 

Standards and test solutions used were purchased from Sigma-Aldrich, Germany. Alkaloid standards prepared in stock solutions were used for vincamine (tr = 5.74 min.), eburnamonin (tr = 11.7 min.), 1,2-dehydroaspidospermidine (tr = 18.8 min.), and vincaminorein (tr = 25.6 min.). These reagents were dissolved in methanol at concentrations ranging from 10 µg/mL to 600 µg/mL. The internal standard used was paclitaxel standard solution in methanol, with a retention time of tr = 5 min.

Methanolic macerates have been prepared using 5 g of dry plant material (stems and leaves) of *Vinca minor* that was vigorously mixed with 50 mL of methanol for 4 h. After the extraction was completed, methanol was evaporated and the residue obtained was dissolved in 30 mL chloroform (CHCl_3_). Four successive extractions were performed using 0.5 mol/L HCl aqueous solution (15 mL HCl solution at each extraction). After extraction, ammonia (NH_3_) was used to adjust the pH to 8.2. The final extracts were concentrated, the residue was dissolved in methanol and the solution obtained was brought to a final volume of 25.00 mL in a volumetric flask. 10 µL of the methanol solution was injected into the chromatographic column.

The obtained hydroalcoholic macerates were analyzed via HPLC-DAD. The operating conditions were mobile phase: methanol: water (0.02 mol/L NH_4_Cl, pH 8.2 adjusted with triethylamine), volume ratios 75:25 and flow rates of 0.5 mL/min. The detector wavelength was 254 nm and the injection volume was 10 µL. Quantification was performed using the internal standard method.

Table 5 and Table 6 presents the results regarding statistical parameters for stock solutions containing indole alkaloids. 

#### 4.2.2. Mineral Composition of *Vinca minor* Plant

Determination of mineral composition was performed using AAS. The solutions used for the determination of metals were prepared by mineralization of 0.5 g dried and ground sample in 5 mL of 69% HNO_3_ and 40 mL of deionized water for 1 h and 30 min at 120 °C. The clear solution obtained via filtration was introduced into 50 mL volumetric flasks and filled with deionized water. A Certipur multi-element standard solution purchased from Merck (1 mg/mL of each metal) was used for calibration. To ensure the quality of the analytical data, the following performance parameters were determined: concentration range (µg/L) and calibration curve correlation coefficients (R^2^), limits of detection (LOD), limits of quantification (LOQ) (Table 7).

The concentration of each metal (mg/kg sample) was calculated with Equation (1):

(1) $$\mathrm{Metal}\;\mathrm{concentration}\;(\mathrm{mg}/\mathrm{kg}\;\mathrm{sample})=\frac{V_{b}\times c}{m_{\mathrm{sample}}}$$

where Vb represents the volume of the volumetric flask in which the sample solution was prepared (50 mL), c represents the concentration of the metal measured in mg/L, and m represents the mass of the sample added to the solution (m = 0.5 g).

#### 4.2.3. Antioxidant Activity Analysis

The antioxidant capacity of *Vinca minor* plant macerates via DPPH radical scavenging test was evaluated using the method described by Stanciu et al. [17]. The calibration curve with gallic acid as standard was linear in the range 0.68–4.76 mg GAE/L and the correlation coefficient was 0.9973. To quantify antioxidant activity, 1 mL of each sample was added to 25 mL calibrated flasks over which 5 mL of 1.268 mM DPPH in methanol was added. The flasks were filled up to the mark with methanol and left in a dark place for 45 min to reach room temperature before recording the absorbance at 530 nm using methanol as a blank [22,23]. Each sample was performed in triplicate, and the average of the results is shown in Table 3.

Determination of the total antioxidant capacity of lipid-soluble substances (ACL) of hydroalcoholic macerates obtained from leaves and stems of *Vinca minor* was performed via the photochemiluminescence method [24]. For the analysis, each hydroalcoholic macerate was diluted in molar ratios of 1:10, 1:100 and 1:200, respectively, with the kit reagent R1 according to the ACL procedure (Analytik Jena AG). Aliquots of 5 μL were taken from each sample (from the supernatant) and exposed to external radiation produced by a phosphor-lined Hg lamp, which provides maximum energy at a wavelength of 351 nm, in the presence of a photosensitive reagent, producing free superoxide anion radicals in the analyzed sample and a photochemical reaction. The free radicals were partially eliminated by the antioxidants present in the sample. Residual radical luminescence was measured as an electrical signal for 120 s and converted to concentration values. The standard reagent kit (Analytik Jena, Germany) of the ACL procedure was used for the analyses: R1 (dilution solvent), R2 (buffer reagent), R3 (photosensitive reagent), R4 (s reagent sized). Mixtures were prepared as shown in Table 8.

**Figure 4:**
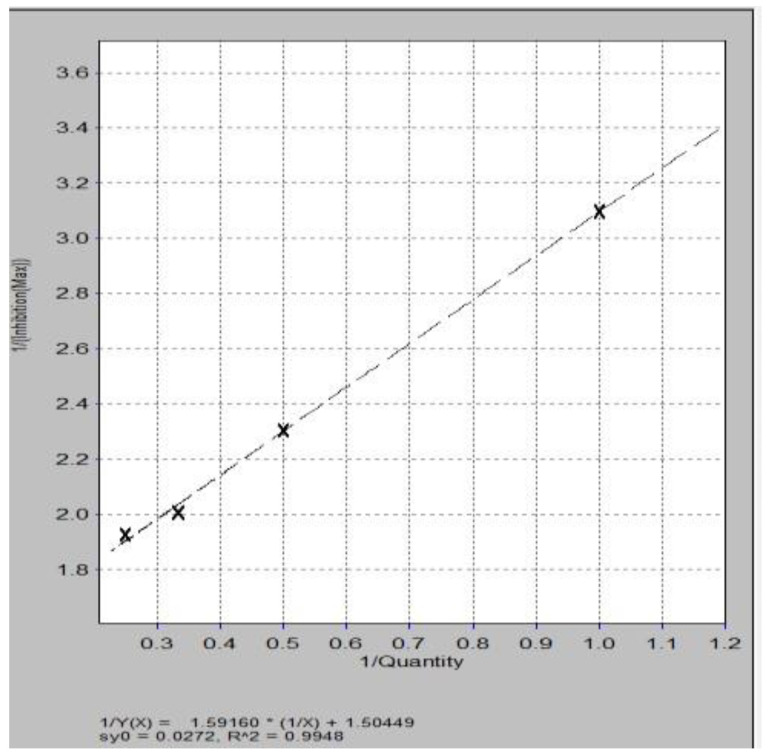
Calibration curve for Trolox standard.

The calibration curve (Figure 4) was calculated using a series of standard solutions containing 0.5, 1.0, 2.0, 3.0 nM Trolox (6-hydroxy-2,5,7,8-tetramethylchroman-2-carboxylic acid), a vitamin E derivative. Determinations were performed in triplicate and were expressed as nM Trolox equivalents (TE)/µL sample, respectively mg (TE)/100 g d.w. sample [25,26,27].

### 4.3. Working Equipment

#### 4.3.1. HPLC Method

HPLC determinations were performed using an Agilent 1260 Infinity HPLC (Agilent, Baden-Württemberg, Germany) provided with a diode array detector and autosampler. Separation and quantification were performed on a C8-type column (250 × 4.6 mm, particle size: 5 μm), Phenomenex, Agilent Co., Santa Clara, CA.

All modules were instrumented with ChemStation HPLC data-acquiring software. The chromatographic data software used for this analysis was Agilent OpenLab CDS ChemStation Edition C.01.05.

#### 4.3.2. Mineral Composition of *Vinca minor* Plant

The ContrAA-700 atomic absorption spectrometer, Analytik Jena AG, Germany, was used to determine the mineral concentration.

#### 4.3.3. Antioxidant Activity Analysis

For the determination of antioxidant activity using DPPH and ACL methods, the following equipment was used: Jasco 550 UV-VIS double phase spectrophotometer using 1 cm layer quartz cuvettes and Photochem, Analytik Jena AG, Germany.

## 5. Conclusions

*Vinca minor* is an important source of valuable compounds used therapeutically to maintain health. In this study, the chemical composition of the plant, *Vinca minor*, was correlated with the antioxidant activity and the analysis of the toxic potential of this plant (mineral composition analysis).

The content of alkaloids present in *Vinca minor* was determined from hydroalcoholic macerates prepared from the leaves and stems of this plant, via the HPLC method. The results showed vincamine to be the predominant alkaloid, followed by 1,2-dehydroaspidospermidine and vincaminoreine. As for the alkaloid eburnamonine, it was found only in plant extracts from the leaves.

The results regarding the concentration of toxic metals below the detection limit confirm the possibility of using *Vinca minor* from the Dobrogea area for therapeutic purposes. The ranking of concentrations of the minerals in the studied plant is Ca > Na > Mg > Fe > Mn > Zn > Cu.

Antioxidant activity of *Vinca minor* hydroalcoholic macerates was evaluated via a DPPH radical scavenging test and the photochemiluminescence method. The results showed high values ranging from 737.626–1123.500 mg GAE/100 g d.w (DPPH assay) and 77.439–187.817 mg TE/100 g d.w (photochemiluminescence method). The different values obtained for leaves and stem macerates are due to the complex and different chemical compositions, depending on the plant material studied and also on the concentration of the solvent used for extraction. The most effective extraction formulation was found to be 70% ethyl alcohol. These results are in agreement with previous measurements of total phenolic content (TPC) and demonstrate a strong correlation between antioxidant activity and the presence of phenolic compounds in macerates.

Following the analysis of the chemical composition associated with the antioxidant potential and the safety obtained in the toxicity profile, *Vinca minor* from Dobrogea can be classified as a plant product with significant potential in the pharmaceutical industry and can be used in the formulation of dermal preparations.

## Figures and Tables

**Table 1:** Results obtained with chromatographic analysis performed on leaf and stem samples of *Vinca minor*.

	Alkaloids	T_r_ Min.	k’	Alkaloid Content	
				Leaf mg/100 g d.w.	Stem mg/100 g d.w.
1	Vincamine	5.74	2.67	2.459 ± 0.035	0.794 ± 0.030
2	Eburnamonin	11.7	5.71	0.803 ± 0.010	-
3	1,2-dehydroaspidospermidine	18.8	10.06	0.898 ± 0.010	1.625 ± 0.034
4	Vincaminorein	25.6	19.94	1.064 ± 0.040	0.285 ± 0.010

d.w. = dry weight.

**Table 2:** Mineral concentration (mg/kg) in *Vinca minor* L.

Metals	Concentration (mg/kg) ± SD	
	Stem	Leaf
Ca	4896 ± 0.52	25,640 ± 3.14
Mg	871.4 ± 0.33	1436.2 ± 1.55
Na	1293.2 ± 0.61	874.20 ± 1.22
Fe	192.34 ± 0.55	620.80 ± 3.27
Mn	92.34 ± 0.74	296.00 ± 1.33
Cd	<DL	<DL
Cu	8.65 ± 0.25	1.40 ± 0.11
Pb	<DL	<DL
Ni	<DL	<DL
Zn	40.8 ± 0.44	64.85 ± 0.14

SD—standard deviation; DL—detection limit.

**Table 3:** Antioxidant activity of hydroalcoholic macerates from leaves and stems of *Vinca minor* plant.

No	Sample	DPPH mg GAE/100 g d.w.
1	F40	1123.500
2	F70	1186.500
3	F96	768.625
4	T40	981.750
5	T70	994.875
6	T96	737.626

d.w.—dry weight.

**Table 4:** Antioxidant activity for diluted solutions of 40%, 70% and 96% hydroalcoholic macerates obtained from leaves and stems of *Vinca minor* plant.

No	Sample/Dilution/Working Volume	Free Radicals Max. Inhibition	Total Antioxidant Capacity (nM TE/μL)	TEAC Quantity Means (mg TE/100 g d.w.)
1.	10% stem extract in 40:60 (*v*:*v*) ethanol/stock sol./5 μL; T40	0.978	3.743	187.367
2.	10% stem extract in 40:60 (*v*:*v*) ethanol/dil. with ethanol 1:10/5 μL; T40	0.719	3.606	180.510
3.	10% stem extract in 40:60 (*v*:*v*) ethanol/dil. with ethanol 1:100/5 μL; T40	0.363	3.174	158.884
4.	10% leaf extract in 40:60 (*v*:*v*) ethanol/stock sol./5 μL; F40	0.995	3.750	187.717
5.	10% leaf extract in 40:60 (*v*:*v*) ethanol/dil. with ethanol 1:100/5 μL; F40	0.226	2.769	138.610
6.	10% stem extract in 70:30 (*v*:*v*) ethanol/stock sol./5 μL; T70	0.972	3.741	187.267
7.	10% stem extract in 70:30 (*v*:*v*) ethanol/dil. with ethanol 1:100/5 μL; T70	0.183	2.567	128.498
8.	10% leaf extract in 70:30 (*v*:*v*) ethanol/stock sol./5 μL; F70	0.999	3.752	187.817
9.	10% leaf extract in 70:30 (*v*:*v*) ethanol/dil. with ethanol 1:100/5 μL; F70	0.724	3.609	180.659
10.	10% leaf extract in 70:30 (*v*:*v*) ethanol/dil. with ethanol 1:200/5 μL; F70	0.107	2.014	100.81
11.	10% stem extract in 96:4 (*v*:*v*) ethanol/stock sol./5 μL; T96	0.960	−3.186	-
12.	10% stem extract in 96:4 (*v*:*v*) ethanol/dil. with ethanol 1:100/5 μL; T96	0.407	1.547	77.439
13.	10% leaf extract in 96:4 (*v*:*v*) ethanol/stock sol./5 μL; F96	0.992	−2.973	-
14.	10% leaf extract in 96:4 (*v*:*v*) ethanol/dil. with ethanol 1:100/5 μL; F96	0.515	3.380	169.196

d.w. = dry weight.

**Table 5:** Statistical parameters regarding calibration curve and limits of detection and quantification for standard solutions of indole alkaloids.

	Alkaloids	Regression Equation	Correlation Coefficient	LOD μg/mL	LOQ μg/mL
1	Vincamine	y = 516.11x − 322.92	0.9998	0.18	0.61
2	Eburnamonin	y = 22,034x + 67,769	0.9998	0.044	0.147
3	1,2-dehydroaspidospermidine	y = 793.05x − 844.26	0.9997	0.17	0.57
4	Vincaminorein	y = 933.31x − 659.68	0.9998	0.01	0.036

**Table 6:** Statistical parameters on precision, accuracy, and repeatability for standard solutions of indole alkaloids.

	Alkaloids	Precision (RSD%)	Accuracy (Standard Deviation %)	Repeatability (R%)
1	Vincamine	0.555	3.13	1.57
2	Eburnamonin	0.535	3.25	1.51
3	1,2-dehydroaspidospermidine	0.789	4.5	2.24
4	Vincaminorein	0.589	3.39	1.67

**Table 7:** Performance parameters for AAS measurements.

Metals	Concentration Range (mg/L)	R^2^	LOD (mg/L)	LOQ (mg/L)
Cadmium	0.050–1.000	0.9938	0.0099	0.0693
Calcium	40.00–200.00	0.9990	84.13	116.1
Copper	0.050–2.000	0.9991	0.0533	0.0586
Iron	0.050–2.000	0.9995	0.0473	0.1183
Lead	0.200–8.000	0.9996	0.1371	1.0280
Magnesium	1.000–5.000	0.9932	0.6440	2.2070
Manganese	0.050–2.000	0.9953	0.0099	0.0597
Nickel	0.100–4.000	0.9969	0.0233	0.1051
Sodium	5.000–25.000	0.9966	1.282	4.669
Zinc	0.050–1.000	0.9922	0.0430	0.1076

**Table 8:** Working scheme of ACL procedure.

Reagents Kit	R1 (μL)	R2 (μL)	R3 (μL)	R4 (μL)	Sample (μL)
Blank	2300 µL	200 µL	25 µL	0 µL	0 µL
Calibration curve	2300	200 µL	25 µL	5 µL	0 µL
Measurement Sample	2300	200 µL	25 µL	0 µL	5 µL

## Data Availability

Not applicable.

## References

[1] Cordell G.A., Quinn-Beattie M.L., Farnsworth N.R. (2001). The potential of alkaloids in drug discovery. Phytother. Res..

[2] Moudi M., Go R., Yien C.Y., Nazre M. (2013). Vinca alkaloids. Int. J. Prev. Med..

[3] Van Vuuren R.J., Visagie M.H., Theron A.E., Joubert A.M. (2015). Antimitotic drugs in the treatment of cancer. Cancer Chemother. Pharmacol..

[4] Karpus I.P. (1961). Morpho-anatomical studies on *Vinca minor* L. from the family Apocynaceae. Farmatsevtychnyi Zhurnal.

[5] Panneerselvam C., Murugan K., Kovendan K., Kumar P.M., Ponarulselvam S., Amerasan D., Subramaniam J., Hwang J.S. (2013). Larvicidal efficacy of Catharanthus roseus Linn. leaf extract and bacterial insecticide Bacillus thuringiensis against Anopheles stephensi Liston. Asian Pac. J. Trop. Med..

[6] Verma P., Khan S.A., Mathur A.K., Shanker K., Lal R.K. (2014). Regulation of vincamine biosynthesis and associated growth promoting effects through abiotic elicitation, cyclooxygenase inhibition, and precursor feeding of bioreactor grown *Vinca minor* hairy roots. Appl. Biochem. Biotechnol..

[7] Adizov S.M., Tashkhodzhaev B., Bruskov V.P., Talipov S.A., Yuldashev P.K., Malikov V.M. (2017). On nitrogen coordination in vincadifformine-type alkaloids. Chem. Nat. Compd..

[8] Cheng G.-G., Zhao H.-Y., Liu L., Zhao Y.-L., Song C.-W., Gu J., Sun W.-B., Liu Y.-P., Luo X.-D. (2016). Non-alkaloid constituents of *Vinca major*. Chin. J. Nat. Med..

[9] Brunke E., Hammerschmidt F., Schmaus G. (1993). Flower Scent of Some Traditional Medical Plants. Bioactive Volatile Compounds from Plants. Am. Chem. Soc. Symp. Wash..

[10] Istudor V. (1998). Farmacognozie. Fitochimie. Fitoterapie. Vol. I. Bucureşti. Ed. Medicală.

[11] Tămaş M. (1999). Botanică Farmaceutică.

[12] Verma P., Khan S.A., Masood N., Manika N., Sharma A., Verma N., Luqman S., Mathur A.K. (2017). Differential rubisco content and photosynthetic efficiency of rol gene integrated *Vinca minor* transgenic plant: Correlating factors associated with morpho-anatomical changes, gene expression and alkaloid productivity. J. Plant Physiol..

[13] Hasa D., Perissutti B., Cepek C., Bhardwaj S., Carlino E., Grassi M., Invernizzi S., Voinovich D. (2013). Drug salt formation via mechanochemistry: The case study of vincamine. Mol. Pharm..

[14] Pu H., Jiang H., Chen R., Wang H. (2014). Studies on the interaction between vincamine and human serum albumin: A spectroscopic approach. Luminescence.

[15] Montvale N.J., Fleming (2004). PDR for Herbal Medicines.

[16] Ciorîță A., Zăgrean-Tuza C., Moț A.C., Carpa R., Pârvu M. (2021). The Phytochemical Analysis of *Vinca* L. Species Leaf Extracts Is Correlated with the Antioxidant, Antibacterial, and Antitumor Effects. Molecules.

[17] Stanciu G., Aonofriesei F., Lupsor S., Oancea E., Mititelu M. (2023). Chemical Composition, Antioxidant Activity, and Antibacterial Activity of Black Poplar Buds’ Hydroalcoholic Macerates from Dobrogea Area. Molecules.

[18] White P.J., Broadley M.R. (2009). Biofortification of crops with seven mineral elements often lacking in human diets--iron, zinc, copper, calcium, magnesium, selenium and iodine. New Phytol..

[19] Pytlakowska K., Kita A., Janoska P., Połowniak M., Kozik V. (2012). Multi-element analysis of mineral and trace elements in medicinal herbs and their infusions. Food Chem..

[20] Mahmood A., Rashid S., Malik R.N. (2013). Determination of toxic heavy metals in indigenous medicinal plants used in Rawalpindi and Islamabad cities, Pakistan. J. Ethnopharmacol..

[21] Dumitrescu A.M., Stanciu G., Sirbu R., Busuricu F. (2021). Spectrophotometric Studies of Indolic Compounds from *Vinca minor* L. Eur. J. Nat. Sci. Med..

[22] Stanciu G., Aonofriesei F., Lupsor S., Popescu A., Sirbu R. (2019). Study of Phenolic Compounds and Antimicrobial Activity of *Lavandula angustifolia* L. Flowers Macerates. Rev. Chim..

[23] Lupsor S., Rotariu R., Oancea E., Oancea I.-A. (2019). Quantitative Analysis of Polyphenols and Antioxidant Activity of Mint Macerate. J. Sci. Arts.

[24] Popov I., Lewin G., Packer L. (1999). Methods in Enzymology, 300, Part B. Oxidants and Antioxidants.

[25] Artem V., Negreanu–Pirjol T., Ranca A., Ciobanu C., Bratu M.M., Popoviciu D.R., Moldovan L., Vasile M., Negreanu-Pirjol B.-S. (2021). Total phenolic content correlated with antioxidant activity of some grape pomace biomass hydroalcoholic extracts, white and red varieties. UPB Sci. Bull. Ser. B Chem. Mater. Sci..

[26] Negreanu-Pîrjol B.S., Negreanu T., Bratu M.M., Roncea F., Mireşan H., Sanda J., Paraschiv G.M., Popescu A. Antioxidative activity of indigen bitter cherry fruits extract corellated with polyphenols and minerals content. Proceedings of the 14th International Multidisciplinary Scientific GeoConferences, Surveying Geology & Mining Ecology Management—SGEM 2014.

[27] Negreanu-Pîrjol B.S., Cadar E., Sirbu R., Negreanu-Pirjol T. (2021). Antioxidant Activity of Some Fluids Extracts of Indigenous Wild Cherry Fruits. Eur. J. Nat. Sci. Med..

